# Conversational Agents in Health Care: Scoping Review and Conceptual Analysis

**DOI:** 10.2196/17158

**Published:** 2020-08-07

**Authors:** Lorainne Tudor Car, Dhakshenya Ardhithy Dhinagaran, Bhone Myint Kyaw, Tobias Kowatsch, Shafiq Joty, Yin-Leng Theng, Rifat Atun

**Affiliations:** 1 Family Medicine and Primary Care Lee Kong Chian School of Medicine Nanyang Technological University Singapore Singapore; 2 Department of Primary Care and Public Health, School of Public Health Imperial College London London United Kingdom; 3 Future Health Technologies programme Campus for Research Excellence and Technological Enterprise (CREATE) Singapore-ETH Centre Singapore; 4 Center for Digital Health Interventions Department of Management, Technology, and Economics ETH Zurich Zurich Switzerland; 5 Center for Digital Health Interventions Institute of Technology Management University of St Gallen St Gallen Switzerland; 6 School of Computer Sciences and Engineering Nanyang Technological University Singapore Singapore; 7 Centre for Healthy and Sustainable Cities Nanyang Technological University Singapore; 8 Department of Global Health and Population, Harvard T.H. Chan School of Public Health Harvard University Boston, MA United States

**Keywords:** conversational agents, chatbots, artificial intelligence, machine learning, mobile phone, health care, scoping review

## Abstract

**Background:**

Conversational agents, also known as chatbots, are computer programs designed to simulate human text or verbal conversations. They are increasingly used in a range of fields, including health care. By enabling better accessibility, personalization, and efficiency, conversational agents have the potential to improve patient care.

**Objective:**

This study aimed to review the current applications, gaps, and challenges in the literature on conversational agents in health care and provide recommendations for their future research, design, and application.

**Methods:**

We performed a scoping review. A broad literature search was performed in MEDLINE (Medical Literature Analysis and Retrieval System Online; Ovid), EMBASE (Excerpta Medica database; Ovid), PubMed, Scopus, and Cochrane Central with the search terms “conversational agents,” “conversational AI,” “chatbots,” and associated synonyms. We also searched the gray literature using sources such as the OCLC (Online Computer Library Center) WorldCat database and ResearchGate in April 2019. Reference lists of relevant articles were checked for further articles. Screening and data extraction were performed in parallel by 2 reviewers. The included evidence was analyzed narratively by employing the principles of thematic analysis.

**Results:**

The literature search yielded 47 study reports (45 articles and 2 ongoing clinical trials) that matched the inclusion criteria. The identified conversational agents were largely delivered via smartphone apps (n=23) and used free text only as the main input (n=19) and output (n=30) modality. Case studies describing chatbot development (n=18) were the most prevalent, and only 11 randomized controlled trials were identified. The 3 most commonly reported conversational agent applications in the literature were treatment and monitoring, health care service support, and patient education.

**Conclusions:**

The literature on conversational agents in health care is largely descriptive and aimed at treatment and monitoring and health service support. It mostly reports on text-based, artificial intelligence–driven, and smartphone app–delivered conversational agents. There is an urgent need for a robust evaluation of diverse health care conversational agents’ formats, focusing on their acceptability, safety, and effectiveness.

## Introduction

### Background

Conversational agents or chatbots are computer programs that simulate conversations with users [1]*.* They are increasingly adopted in many different fields, including finance, commerce, marketing, retail, and fitness, with favorable reception from customers [2]. Conversational agents are often deployed via messaging apps, a website, or a mobile phone app. They can also be integrated into cars and television sets or in the form of a stand-alone device such as speakers. They can converse through a range of methods such as text, image, and voice. Conversational agents that can interpret human speech and respond via synthesized voices as well as manage tasks requested by the user are also known as voice assistants. Some of the most popular voice assistants include Apple’s Siri, Amazon’s Alexa, Google Assistant, and Microsoft’s Cortana, mostly delivered using voice-activated or smart speakers such as Amazon’s Echo and Google Home. They are utilized for aiding or executing tasks such as web-based shopping, control of smart home devices, and disseminating news or for entertainment [3-5].

Conversational agents cover a broad spectrum of aptitudes ranging from *simple* to *smart* [2]. Simple conversational agents are *rule based*, meaning that they depend on prewritten keywords and commands programmed by the developer. The user is therefore restricted to predetermined options when answering questions posed by the conversational agents, and there is little or no opportunity for free responses. If a user enters a question or sentence without a single keyword, the conversational agents will be unable to understand the input and will respond with a default message such as “Sorry, I did not understand” [2]. Despite these restrictions, simple conversational agents are increasingly used in executing tasks such as booking appointments, purchasing merchandise, ordering food, and sharing information without the need for human involvement [2].

In contrast, smart conversational agents do not respond with preprepared answers but with adequate suggestions instead. This is enabled by machine learning, a type of artificial intelligence (AI), which allows for broadening of the computer system’s capacity through its learning from data (in this case conversations) without being explicitly programmed [2,6]. The process whereby the machine translates human commands into a form in which the computer can understand, process, and revert to the user is called natural language processing (NLP) [6] and natural language understanding or interpretation [6,7]. This degree of programming allows for personalized conversational agents to be generated. Smart conversational agents have the potential to undertake more complex tasks that involve greater interaction, reasoning, prediction, and accuracy. Although the technology behind smart conversational agents is continuously developed, they currently do not have full human-level language abilities, resulting in misunderstanding and users’ dissatisfaction [8]. Furthermore, as machine learning algorithms develop, it is becoming increasingly challenging to keep track of their development, evolution, and the reasoning behind their responses. This is known as the *black box effect* [9,10]. Although the black box effect appears to be an unavoidable consequence of the use of AI, there is some emerging research on making AI transparent and explainable [11]. However, at the moment, its use may affect the safety and accuracy of treatment and should be carefully monitored and evaluated when used in health care [9].

**Figure 1:**
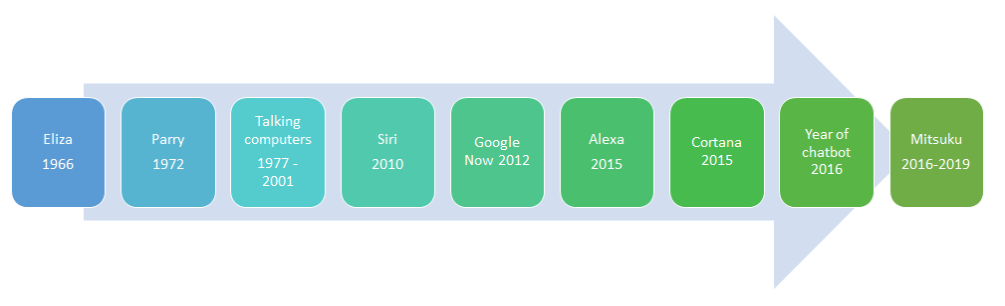
Evolution of conversational agents from 1966 to 2019.

The first conversational agent *ELIZA* was developed by Weizenbaum [12] in 1966, with ELIZA taking on the role of a person-centered Rogerian psychotherapist (Figure 1). This was a groundbreaking contribution to the field of AI and was reported to have a positive impact on patients who communicated with the conversational agent [13]. A step up from ELIZA was achieved when *PARRY*, a conversational agent representing a simulated paranoid patient with schizophrenia, was developed [14,15]. These first examples of conversational agents, *chatterbots* (as they were referred to then), in health care were valuable in demonstrating that virtual agents have the potential to mimic human-human conversation and successfully pass the Turing Test, a test of a machine’s ability to replicate human intelligence, and the machine passes the test when the tester cannot distinguish it from the human [16].

The literature over the next few decades does not explicitly mention *chatbots* or *conversational agents* in health care, but it does refer to *talking computers* [17-21], a less sophisticated version of today’s conversational agents previously used for conducting patient satisfaction surveys [17], altering adult eating habits [18], aiding health care service delivery through diagnosis aid [19], and promoting patient-physician communication [20]. Although not presented in the literature, chatbot Jabberwacky was released in 1988. It was one of the first few AI agents developed for human interaction and entertainment and introduced the shift from text- to voice-operated conversational agents. Soon after, ALICE gained plenty of attention in 1995, after which it went on to win the Loebner Prize 3 times in 2000, 2001, and 2004.

The next big milestone for conversational agents was in 2010 when Apple released *Siri*. The interest in conversational agents increased exponentially at this point as evidenced by Google, Amazon, and Microsoft all developing their own versions over the coming years: Google now, Alexa, and Cortana, respectively [14]. Year 2016 was named the *Year of the Chatbot* as a number of major information technology companies started to use conversational agents: Facebook launched its messenger platform for conversational agents, Google announced its procurement of the conversational agent development tool API.ai, LinkedIn revealed its first messaging bot, and Viber released Public Accounts for chatting with businesses [22-25]. Currently, the title of the world’s best conversational agent is held by Mitsuku, a 4-time winner of the Loebner Prize, an annual competition in AI [26].

Health care, which has seen a decade of text messaging on smartphones, is an ideal candidate for conversational agent–delivered interventions. Conversational agents enable interactive, 2-way communication, and their text- or speech-based method of communication makes it suitable for a variety of target populations, ranging from young children to older people. The concept of using mobile phone messaging as a health care intervention has been present and increasingly explored in health care research since 2002 [27]. A series of systematic reviews on the use of text messaging for different health disorders have shown that text messaging is an effective and acceptable health care intervention [28,29]. With a global penetration rate of 96% [28], mobile phones are ubiquitous and avidly used, and can be efficiently harnessed in health care [30]. Conversational agents are increasingly used in diverse fields, including health care, and there is a need to identify different ways and outcomes of the use of conversational agents in health care. Existing reviews on conversational agents focus on a certain subtype of agents such as virtual coaches [31-33] or embodied conversational agents (ECAs) [34] or on specific functionalities of these agents such as behavior change [35] or mental health applications [36,37]. Other reviews report solely on the technical aspects of conversational agents such as system architecture and dialogues [38] or on the funding component of health care conversational interfaces [39].

### Objectives

Our objective was to provide a comprehensive overview of the existing research literature on the use of health care–focused conversational agents. We aimed to examine how conversational agents have been employed and evaluated in the literature to date and map out their characteristics. Finally, in line with the observed gaps in the literature, we sought to provide recommendations for future conversational agent research, design, and applications.

## Methods

### Search Strategy

We adopted methodological guidance from an updated version of the Arksey and O’Malley framework with suggestions proposed by Peters et al [40] in 2015 to conduct our scoping review. To identify literature pertaining to the application of conversational agents in health care, a broad literature search was conducted in April 2019 in MEDLINE (Medical Literature Analysis and Retrieval System Online; Ovid), EMBASE (Excerpta Medica database; Ovid), PubMed, Scopus, and Cochrane Central. Given the novelty of the field, the amount of ongoing research happening in the area, and to increase comprehensiveness, we also searched for the gray literature in the OCLC WorldCat database, ResearchGate, Google Scholar, OpenGrey, and the first 10 pages of Google.

We used an extensive list of 63 search terms, including various synonyms for conversational agents (Multimedia Appendix 1). These synonyms were generated using a web-based search and by identifying specific terms or phrases used in the titles of articles discussing health care conversational agents. The reference list of relevant articles and systematic reviews were also searched for further articles related to the review.

### Inclusion and Exclusion Criteria

To map out the current conversational agent applications in health care, we included primary research studies that had conducted an evaluation and reported findings on a conversational agent implemented for a health care–specific purpose. We excluded articles that just presented a proposal for conversational agent development, articles that mentioned conversational agents briefly or as an insignificant part of a review, as well as opinion pieces and articles where primary research was not conducted or discussed. A further point of exclusion was articles with poorly reported data on chatbot assessments where there was minimal or no evaluation data. In addition, we excluded articles concerning ECAs, relational agents, animated conversational agents, or other conversational agents with a visual or animated component.

ECAs are computer-generated virtual individuals with an animated appearance to enable face-to-face interaction between the user and the system [41]. Relational agents are a type of ECA designed to create long-term deep and meaningful relationships with individuals [42]. ECAs are similar to conversational agents in that conversation is central to their function; however, ECAs are more complex as hand movements and facial expressions can be conveyed to the user as well [41]. The user’s interaction may be affected by nonverbal behaviors, graphics, and layout of the program, and it was decided that the complexities associated with ECAs are beyond the scope of this review and were therefore excluded.

### Screening, Data Extraction, and Analysis

Screening of articles for inclusion was performed in 2 stages: title and abstract review and full article review, undertaken independently by 2 reviewers. Following an initial screening of titles and abstracts, full texts were obtained and screened by 2 reviewers. From the included studies, 2 reviewers independently extracted relevant information in an Excel (Microsoft) spreadsheet. We extracted data on the first author, year of publication, source of literature, title of article, type of literature, study design and methods, geographic focus, health care sector, conversational agent name, accessibility of conversational agent, dialogue technique, input and output modalities, and nature of conversational agent’s end goal. We piloted the data extraction sheet on at least five articles. Potential discrepancies in the extracted data were discussed between the authors and resolved through discussion and consensus.

We performed a narrative synthesis of the included literature and presented findings on (1) study specifics, such as study design, geographic focus, and type of literature; (2) conversational agent specifics (ie, conversational agent delivery channel, dialogue technique, personality, etc); (3) conversational agent content analysis; and (4) study evaluation findings.

We used the principles of thematic analysis to analyze the content, scope, and personality traits of the conversational agents. Two researchers familiarized themselves with the literature identified, generated the initial codes in relation to personality and content analysis, applied the codes to the included studies, compared their findings, and resolved any discrepancies via discussion.

The need to present information on conversational agent personality was motivated by the concepts presented in the study by de Haan et al [43], which posits that personalities are not just limited to humans but can be extended to nonhuman artifacts to explain their actions and behavior [43]. Furthermore, it states that personality traits are especially important in the design of *socially interactive robots*, such as conversational agents. The 5 dimensions of personality presented in this paper were derived from the following: extraversion, agreeableness, conscientiousness, emotional stability, and culture. We have used these headings to guide our analysis of the conversational agents’ personality traits in this review. We also aimed to identify and analyze the patterns in the description of conversational agents pertaining to personality traits. Multiple codes were sometimes assigned to the same agent where necessary, but this was limited to a maximum of 3 codes to maintain some degree of specificity.

## Results

### Search Findings

**Figure 2:**
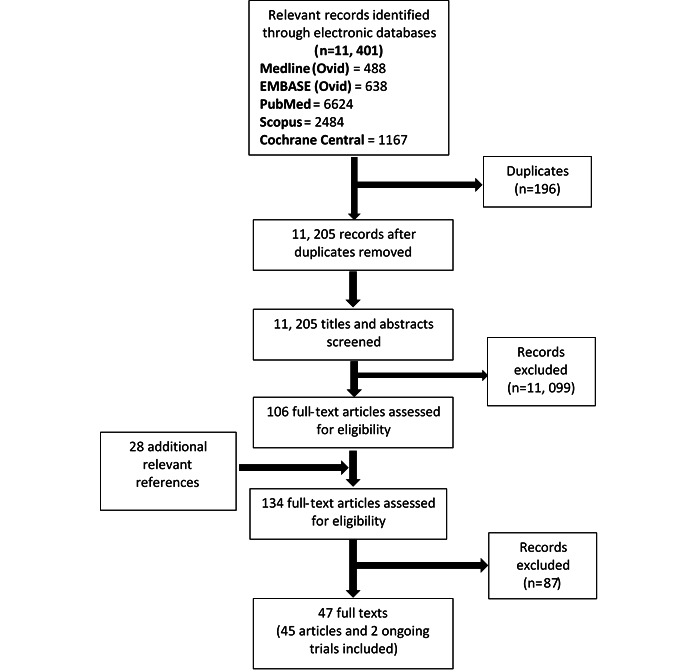
PRISMA flow chart.

The initial database searches yielded 11,401 records, and another 28 records were retrieved through additional sources such as the gray literature sources and screening of reference lists of relevant studies. A total of 196 duplicates were identified and removed, leaving 11,233 titles and abstracts that needed to be screened. Title and abstract screening led to the exclusion of 11,099 records, resulting in 134 full texts that needed to be assessed for eligibility. Of these, 87 articles were excluded, resulting in a final pool of 47 reports comprising 45 studies and 2 ongoing trials (Figure 2).

### Characteristics of Included Studies

In this scoping review, 40 included studies were from high-income countries (HICs) and 6 were from low- and middle-income countries (LMICs). A total of 22 studies were from European countries, including Italy [44,45], Switzerland [30,46-52], France [53,54], Portugal [55], The Netherlands [56], the United Kingdom [57-61], Spain [62,63], and Sweden [64]. Moreover, 8 studies originated from Asian countries: Philippines [65], China [66], Japan [67,68], Pakistan [69], India [70,71], and Hong Kong [72]. Other geographic regions acknowledged in the studies of this review were Australia [73,74], Canada [75], New Zealand [76,77], South Africa [78], and the United States of America [79-89].

**Figure 3:**
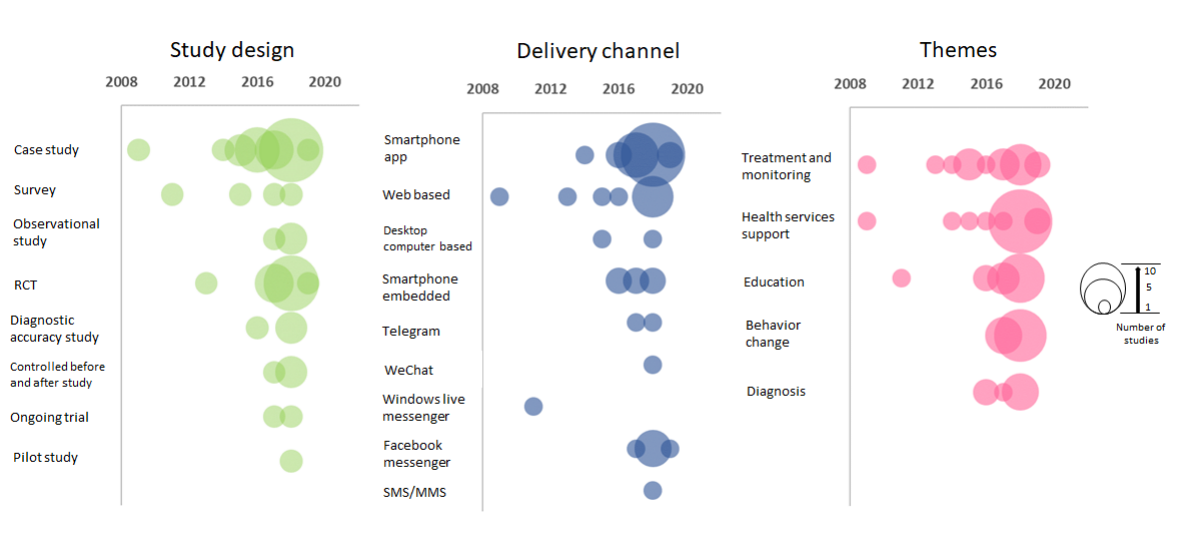
Bubble plots showing the distribution of identified study designs, types of conversational agents and healthcare topics in the included articles, plotted against the year of the publication. The scale on the right indicates that the size of the bubble is associated with the number of studies whereby the smallest denotes 1 study and the largest, 10 studies.

A variety of study designs were used in the included studies, comprising 20 case studies [44,48,51,61-63,66,69,71, 73-79,82,84,85,89], 4 surveys [55,56,59,65], 3 observational studies [53,86,87], 11 randomized controlled trials [46,49,50,57,64,67,72,80,81,83,88], 3 diagnostic accuracy studies [58,60,68], 3 controlled before and after studies [30,45,70], 2 ongoing trials [51,54], and 1 pilot study [47] (Figure 3).

The types of literature included 25 journal articles [44,48,55-57,61-65,67,69,72,74-76,80-87,89], 11 conference abstracts [45,47,49,50,52,59,70,71,73,78,79], 4 conference papers [30,46,66,77], 1 poster abstract [68], 4 electronic preprints [53,58,60,88], and 2 clinical trial protocols [51,54].

There was an increase in the number of publications each year, from 3 in 2015 to 5 in 2016, 10 in 2017, and 23 in 2018. Some author groups were highly productive and published at least two papers within 2 years. Kowatsch et al published 3 papers between 2017 and 2018 based on their open source behavioral intervention platform MobileCoach, which allows the authors to design a text-based health care conversational agent for obesity management and behavior change [30,46,90]. Griol et al published articles on conversational agent for chronic conditions, including chronic pulmonary disease [63] and Alzheimer disease [62] in 2015 and 2016, respectively. Such productive teams reiterate the research interest in this area of conversational agents. Furthermore, the high frequency of publication indicates the feasibility and support to conduct research successfully in this area.

### Characteristics of Conversational Agents in the Included Studies

#### Conversational Agent Delivery Channel

Conversational agents were delivered through a variety of means in the included studies. Most (n=23) were smartphone apps [30,46-50,53,55,58-61,64,67,70,71,75,77,81,83,85,86,88]; web based (n=5) [57,66,73,74,82]; desktop computer based (n=2) [65,79]; used smartphone-embedded software (n=6; eg, Siri, Google Assistant, Alexa, etc) [44,51,62,76,84,87], Telegram [45,78], WeChat [72], SMS and multimedia messaging service [89], Windows live messenger [56], or Facebook Messenger [52,80]; and 4 were made available on more than 1 platform [53,59,68,83]. Three studies did not specify the method of conversational agent delivery [54,63,69].

#### Technical Development Approach

A total of 8 studies made a reference to the technical details of the conversational agent development process. Some mentioned specific tools such as C and MS Access [65]. Others discussed the application of well-known concepts, to conversational agent development such as using the Computers are Social Actors paradigm to develop a health advice conversational agent, or converting the structure association technique (SAT) into digital SAT for implementation on a LINE platform [67,83]. Some emphasized data set creation and sources for the knowledge base [44]. Four studies provided an in-depth workflow with a step-by-step explanation of the technical development of the conversational agent. Cheng et al [79] provided a very detailed technical explanation of the development process—broken down and explained in parts: program development on Google’s home device, webhook and internal logic, and web interface. Galescu et al [82] described the CARDIAC system architecture including a knowledge base, task models, dialogue management, speech recognition, and language generation. Griol et al [63] presented a spoken dialogue system with specific details of the proposed emotion recognizer. For example, it considers pitch, frequency, energy, and rhythm of speech input from the user. Joerin et al [75] provided a less technically dense explanation for chatbot conversational agent development but mentioned technologies used in the process, such as emotion algorithms and machine learning techniques [75].

#### Input and Output Modalities

The conversational agents could be categorized according to whether the user input was fixed (ie, predetermined text) or unrestricted (ie, free text/speech). A total of 10 studies employed fixed text user inputs [30,46,47,49,50,52,54,58,83,88], with 2 additional studies enabling fixed text and image inputs [67,68]. Moreover, 19 studies allowed free text user inputs [45,48,51,56,57,60,61,66,69,70,72,74,77,78,80,81,85,86,89], and 4 studies used both fixed and free text user inputs [53,64,65,73]. Speech was enabled in 8 studies [44,55,63,71,76,79,82,84], whereas free text and speech were employed in 3 studies [62,75,87]. The method of user input was unspecified in 1 study [59] (Multimedia Appendix 2).

Similarly, output modalities largely employed text alone (n=30) [45-47,49-51,53,54,56-58,60,61,64-66,68-70,72-74,77,78,80,81,83,85,88,89]; text and speech (n=5) [48,55,63,71,87]; speech alone (n=4) [44,79,82,84]; text and images (n=4) [30,67,75,86]; text, speech, and images [62]; or text, speech, images, and videos [52,76]. The input and output methods were not specified in 1 of the studies [59] (Multimedia Appendix 2).

#### Conversational Agent Personality

We condensed the descriptive terms used in individual studies to present the conversational agents into a list of 9 relevant personality traits as presented in Table 1.

The conversational agents in the included studies were health care professional like [57,58,62,66,71,73,74,86], informal [46,52,53,56,61,65,81,85], coach like [47,49,52,64,66,70,80], knowledgeable [56,60,68,72,89], human like [48,78,79,88], culture specific [47,48,53], factual [68,76], gender specific [46,78], and some identified explicitly as a conversational agent [46,65].

One article [78] reported on a conversational agent personality that was criticized for being overly formal, and some articles did not report on the personality of the conversational agent at all [30,44,45,50,51,54,55,59,63,67,69,75,77,82-84,87].

**Table 1:** Personality codes derived for the conversational agents included in this review, adapted from Haan et al.

Personality codes	Descriptions
Coach like	Encouraging, motivating, and nurturing
Conversational agent identity	Explicitly identifies as a conversational agent
Culture specific	Speaks the native language or has native names
Factual	Nonjudgmental, no personal opinions, and responses based on facts or observations
Gender specific	Male and female versions available
Health care professional like	Designed to be a doctor or expert, that is, mimics a health care professional
Human like	Tries to emulate humans, for example, participants reported feeling like they were talking to another human or researchers used features like “typing” to make the conversation more human like
Informal	Informal, like talking to a friend. Uses exclamations, abbreviations, and emoticons
Knowledgeable	Content created or informed by medical experts

#### Human Involvement

A health care administrator or professional was available via the conversational agent for the user to communicate with in some studies. The role of the human varied from an administrator who could be contacted via a dedicated chat channel for the user to ask questions or an individual whose role was to monitor the user’s activity on the conversational agent and provide personalized feedback to them. Seven studies [30,46,47,70,72,78,85] reported on human involvement in the conversation and the remaining articles did not.

#### Conversational Agent Goals

All the conversational agents in this review were identified as *goal oriented*. Goal-oriented conversational agents have a clearly defined end point and are employed to execute a specific function, unlike *chit chat* agents that have no specific end goal, do not delve into the details of any topic, and have a primary aim of merely keeping the conversation going [91]. Goal-oriented conversational agents were further divided into those that yielded long- or short-term outcomes. Of the included studies, 22 articles focused on conversational agents with long-term goals and 23 with short-term goals (Multimedia Appendix 3 [30,44-89]). Two studies reported on conversational agents with both short-term and long-term goals [45,56], for example, answering immediate queries (short) and providing education and increasing users’ knowledge on the topic over time (long) [56]. Conversational agents with short-term scope provided users with a response or service almost instantaneously, such as answering health-related queries [84]. Conversely, those with long-term scope needed to build a relationship with the user, over time, to help them overcome health-related issues such as smoking cessation [72] or working through a mental health problem [80].

### Conversational Agent Content Analysis

Five distinct themes were identified in terms of conversational agent content: treatment and monitoring (ie, treatment implementation, management, adherence, support, and monitoring), health service support (ie, connecting patients to health care services), education (ie, provision of health care–related information), lifestyle behavior change (ie, supporting users in tackling various modifiable health risk factors), and diagnosis (ie, identification of the nature of a disease or a condition). A number of included conversational agents spanned several different themes (Multimedia Appendices 3 and 4 [30,44-89]).

#### Treatment and Monitoring

Overall, 17 articles reported on conversational agents that focused on treatment, monitoring, or rehabilitation of patients with specific conditions. One study reported on a conversational agent to help preserve cognitive abilities in those with Alzheimer disease [62]. Two other studies focused on conversational agents to provide support and treatment for metabolic conditions such as type 2 diabetes [70] and obesity [46]. Eight studies presented conversational agents for managing mental health using techniques such as counseling [67]; cognitive behavioral therapy (CBT) [64,80] method of levels therapy [57]; positive psychology [61]; provision of a virtual companion [66]; and a combination of modalities such as CBT with mindfulness-based therapy, emotionally focused therapy, and motivational interviewing [75,81]. One study each reported on the use of a conversational agent for monitoring patients with asthma [85], HIV [45], heart failure [82], and chronic respiratory disease management [63]. Non–disease-specific conversational agents were used as a health information advisor [83] and pediatric generic medicine consultant [65].

#### Health Care Services Support

Overall, 19 studies reported on conversational agents used to support or complement existing health care services. These tasks included remote delivery of health care services for mental health support [67,75,81], breast cancer [53,54], dysarthria [44], obesity [50], diabetes management [79], chronic respiratory diseases [63], asthma [85], heart failure [82], and HIV management [45]. Other studies discussed conversational agents automating health care services such as patient history taking [48,77], providing health advice [83], symptom checking [58], and triaging and diagnosis support [60,69,74].

#### Education

We found 13 articles in which conversational agents were used primarily for educating patients or users. Education focused on topics such as sexual health [59,76] including information on HIV [78], overcoming unhealthy habits such as alcohol misuse [73] and smoking cessation [72], improving well-being [88], diabetes management [79], breast cancer [53,54], and medication-related queries [55] as well as general health [56,84,87], which covered more than 1 topic of focus, for example, education on sex, drugs, and alcohol for adolescents.

#### Lifestyle Behavioral Changes

We identified 12 studies with conversational agents for healthy lifestyle behavior change in the general population as well as overweight and obese individuals. Two studies discussed conversational agents for the management of obesity in younger patients, including adolescents [46,50]. They largely employed a coach-like conversational agent to promote physical activity [51] and healthy eating [52], sometimes with incentive provision, and provided techniques on how to reverse obesity [30,47,49,71]. Other behavioral change interventions used a social media–driven conversational agent for smoking cessation [72], a health coach for diabetes prevention [86], a reflection companion to encourage physical activity in adults [89], and emotionally intelligent agents to improve mental health [61] and well-being [88].

#### Diagnosis

Seven articles presented health care conversational agents with a primary purpose of establishing a diagnosis. Three articles reported on conversational agents’ triage, diagnosis, or a combination of both, mainly employing a symptom checker function [58,60,74]. Three more studies reported purely on the diagnostic accuracy of 2 conversational agents [69,71,77]. One article reported on a conversational agent for diagnosing sexually transmitted infections to overcome barriers such as social stigma, embarrassment, and discomfort associated with traditional diagnostic approaches that require a medical interview with a health care professional [68].

### Conversational Agent Evaluation

Included studies that evaluated conversational agents reported on their accuracy (in terms of information retrieval, diagnosis, and triaging), user acceptability, and effectiveness. Some studies reported on more than 1 outcome, for example, acceptability and effectiveness. In general, evaluation data were mostly positive, with a few studies reporting the shortcomings of the conversational agent or technical issues experienced by users. Seventeen studies presented self-reported data from participants in the form of surveys, questionnaires, etc. In 16 studies, the data were objectively assessed in the form of changes in BMI, number of user interactions, etc. In 12 studies, there was a mixture of self-reported and objectively assessed outcomes and outcomes were not reported in the two ongoing trials (Multimedia Appendix 4).

#### Accuracy: Information, Diagnosis, and Triaging

Eleven studies reported on the accuracy of conversational agents [44,58,60,66,68,69,71,74,76,77,82] (Multimedia Appendix 4). Middleton et al [58] and Razzaki et al [60] evaluated 2 versions of the Babylon conversational agent, respectively: *Babylon check* and *Babylon chatbot for triage and diagnosis*. In both studies, the conversational agents were tested on their triage and diagnostic accuracy using clinical vignettes as in the Membership of the Royal College of General Practitioners exams, and their performance was compared with that of doctors. The conversational agents were found to be more accurate, faster, and provided safer triage and diagnosis compared with doctors and nurses. Similarly, Ghosh et al [74] and Danda et al [71] assessed conversational agents on their general diagnostic accuracy, and these had a precision rate of 82% and 86%, respectively. Ni et al [77] assessed Chatbot MANDY, designed to automate patient intake, on its ability to adequately diagnose the patient based on their symptoms. There was a prediction accuracy of 100%, 64%, 25%, and 14% for respiratory issues, chest pain, headache, and dizziness, respectively [77]. Furthermore, 2 studies tested the accuracy of conversational agents employed for sexual health purposes [68,76]. The conversational agent used by Kobori et al [68] diagnosed sexually transmitted infections with an accuracy of 77.7% and had high effectiveness (97.7%) in encouraging patients to visit the clinic earlier. In contrast, Wilson et al [76] compared smart assistants—Google Assistant, Siri, and Google search—to determine their accuracy in responding to queries around sexual health. The Google search option was found to provide the best answers and also had the lowest failure rate [76]. Another study compared 3 known virtual assistants—Siri, Google Assistant, and Amazon Alexa—on their abilities to recognize speech from individuals with dysarthria [44]. They all performed similarly (50-60% recognition), with Siri being the only agent attempting to parse all the dialogue inputted [44]. Two studies discussed the accuracy of 2 conversational agents in making diagnoses in children and adolescents [66,69]. Teenchat had a 78.34% precision rate in diagnosing stress [66], whereas Aquabot had an accuracy of 85%, 86.64%, and 87.2% (3 groups aged 18-28 years) for achluophobia and 88%, 87.6%, and 87.53% (3 patient groups aged 1-7 years) for autism [69]. Finally, Galescu et al [82] discussed the accuracy of a conversational agent *CARDIAC* in speech recognition for heart failure patients. A significant number of errors were detected and attributed to insufficient vocabulary coverage in the language model as evidenced by an *out-of-vocab* rate of 3% [82].

#### Effectiveness

The effectiveness of health care conversational agents was assessed in 8 studies [47,52,57,61,70,75,81,84]. Furthermore, 10 studies reported on the effectiveness and acceptability, of which 5 are presented here [49,64,67,80,86] and the remainder are presented under *Acceptability* (Multimedia Appendix 4). Five studies described conversational agents targeting a healthy lifestyle change specifically for healthy eating [52], active lifestyle [49], obesity [47], and diabetes management [70,86]. Casas et al [52] reported improvements in food consumption, whereas Stasinaki [47] and Heldt et al [49] noted increases in physical activity performance with high compliance. Shaikh et al [70] reported successful reduction in HbA_1c_ (glycated hemoglobin) levels postengagement with *Wellthy diabetes*, whereas Stein et al [86] reported successful weight loss (2.38%) and satisfaction was high, rated at 87% for the diabetes prevention chatbot.

Eight studies noted the effectiveness of conversational agents for mental health applications [57,61,64,67,75,80,81,84]. The conversational agent *Tess* by Fulmer et al [81] initiated a statistically significant improvement in depression and anxiety compared with the control group. Two studies looked at the use of machine learning–based conversational agents for CBT in young adults [64,80]. The conversational agent was both effective (reduced levels of depression and perceived stress and improved psychological well-being) and well received (high engagement with the chat app and high levels of satisfaction) [64,80]. This positive effect was reproduced by Joerin et al [75]*,* where emotional support from Tess decreased symptoms of anxiety and depression by 18% and 13%, respectively [75]. Inkster et al [61] employed the Patient Health Questionnaire-9 self-reported depression scale to note significant improvements in depression scores in the high user group compared with the low user group [61]. In addition, 67.7% of users found the app usage to be helpful and encouraging [61]. In the study by Kamita et al [67], the counseling bot encouraged significant improvements in users’ self-esteem, anxiety, and depression compared with the control condition. Besides effectiveness, user ratings of acceptability, using the technology acceptance model, were higher in the conversational agent condition compared with the control [67]. Gaffney et al [57] proposed a conversational agent *MYLO* that was significantly better than the existing conversational agent ELIZA in problem solving and helpfulness, but both were equally effective in lowering distress. Miner et al [84] compared Apple’s Siri, Microsoft’s Cortana, Samsung’s S Voice, and Google Now on their abilities to respond to questions about mental health, interpersonal violence, and physical health. Siri responded appropriately and empathetically to issues concerning depression and physical health, and Cortana responded appropriately and empathetically to matters involving interpersonal violence [84].

#### Acceptability

A total of 26 studies commented on the acceptability of conversational agents (Multimedia Appendix 4). Five studies commenting on acceptability and effectiveness were discussed above [49,64,67,80,86] (see the *Effectiveness* section), and the remaining 21 studies are presented here [30,45,46,48,50,53,55,56,59,62,63,65,72,73,78,79,83,85,87-89]. Several studies (n=6) were targeted at children or adolescents. Three studies discussed conversational agents for health education on medication, asthma management, drugs, sex, and alcohol [56,65,85]. Acceptability was generally denoted by high response rates and scores like *strongly agree* or *agree* for user-friendliness, appropriateness, consistency, and speed of response [65]. In addition, users in the study by Crutzen et al [56] favored the conversational agent over existing methods of information provision. In another 3 studies, conversational agents were employed for the management of obesity in adolescents [30,46,50]. Acceptability was high in all studies, as evidenced by enjoyment of the chats; bonding; formation of social and emotional relationships; and high perceived ease of use, usefulness, and intention to use [30,46,50]. In the study by L’Allemand et al [50]*,* high compliance was attributed to the rewarding game system.

In 4 studies, health care conversational agents were targeted at chronic conditions [55,62,63,79]. The specific conditions addressed were Alzheimer disease, diabetes, heart failure, and chronic respiratory disease. In the study by Cheng et al [79], users responded positively, particularly to features of conversational agents that allowed for personalization and the conversational agent’s ability to understand and respond to natural conversation flow. Some difficulties included learning commands, restricted answer options, slow processing speed, and some problematic responses [79]. Lobo et al [55] reported user acceptability in the form of usability, where the conversational agent had a system usability score of 88, which was considered *very good*. Griol et al [62] considered the Alzheimer patients’ caregiver’s perspective when judging the acceptability of the conversational agent. The global rate for the system (on a scale from 0 to 10) was 8.6, and the application was thought to be attractive, adequate, and appropriate for its purpose. In another study, Griol et al [63] employed an emotionally sensitive conversational agent for chronic respiratory disease patients who rated this agent significantly higher for interaction rate, frequency, and empathy than the baseline version.

A further 3 studies were concerned with sexual health and/or HIV management [45,59,78]. They indicated that in this field, conversational agents could be used for a variety of functions such as booking an appointment, getting test results, therapy, and event reminders [45]. In addition, the conversational agent in the study by van Heerden et al [78] was well received when used as a counseling tool because it was given an avatar-like profile image and the conversation was embedded in a familiar chat interface, which users associated with talking to another human being. In the study by Nadarzynski et al [59]*,* users favored the conversational agent because of its ubiquity as a convenient smartphone app and its ability to perform remote services such as video consultation, potentially alleviating any inhibitions users may have around discussing sexual health in person.

Two studies employed an emotionally sensitive conversational agent for mental health counselling and general health information advice [83,88]. In the study by Liu et al [83]*,* the sympathetic conversational agent was rated more positively than the advice-only condition. Another conversational agent for well-being improvement procured positive feedback from participants who thought it was *an interesting experience*, *pretty quick*, and *fun* [88].

In 3 studies, conversational agents were used for healthy behavior change, specifically targeting smoking cessation, alcohol misuse treatment, and physical activity promotion [72,73,89]. For smoking cessation, participants indicated enjoyment when conversing with the conversational agent, and effectiveness was also insinuated by 38.3% reporting not having smoked in the past week and 69.4% admitting to a reduction in smoking frequency [72]. In the study by Elmasri et al [73], the participants (young adults) reported a higher satisfaction rate with the use of the conversational agent to manage and treat alcohol misuse. For physical activity promotion through the use of a reflection companion, response rates were high (96% at baseline, 90% at follow-up), insinuating high engagement throughout the study. Furthermore, use of the system beyond the stipulated study period was an indicator of viability. Moreover, 16 of the 33 participants opted to continue without any reward, suggesting participants found some added value in using the conversational system [89].

Two studies examined the acceptability of conversational agents for health care service delivery [48,87]. Outcomes were reported qualitatively, including comments on ease of use, humanity of the chatbot, and users’ comfort with the input functionalities available to them as well as criticisms on technical difficulties [48]. Bickmore et al [87] more specifically compared conversational assistants Siri, Alexa, and Google Assistant on their provision of health information and found satisfaction to be lowest with Alexa and highest with Siri. Overall, there was a neutral rating for satisfaction, with a median score of 4 (IQR 1-6) [87].

One study discussed a condition-specific conversational agent application targeted at improving the quality of life and medication adherence of breast cancer patients [53]. Participants implied a positive experience when interacting with the conversational agent, whereby 88% said it provided them with support in tracking their treatment and mentioned that they would recommend the conversational agent to their friends. There was an overall satisfaction of 94% [53].

## Discussion

### Principal Findings

Our scoping review identified 45 studies and 2 ongoing clinical trials. Although conversational agents have been widely employed in various fields, their use in health care is still in its infancy, as evidenced by the study findings that indicate much of the literature being published recently (2016-2018). Most conversational agents used text input and were machine learning based and mobile app delivered. The 3 most commonly reported themes in the health care conversational agent–related literature were treatment and monitoring, health services support, and patient education. Results from the studies evaluating conversational agents were generally positive, reporting effectiveness, accuracy, and acceptability of the conversational agent. However, there is currently a dearth of robust evaluations and a predominance of small case studies.

Our review shows that most of the health care conversational agents reported in the literature used machine learning and were long-term goal oriented. This suggests that conversational agents are evolving from conducting simple transactional tasks toward more involved end points such as long-term disease management [80] and behavior change [30]. The majority of the conversational agents identified in this review targeted patients, with only a few aimed at health care professionals, for example, by automating patient intake or aiding in patient triage and diagnosis. In addition, research into the use of conversational agents to support both formal and informal caregivers is limited and could be a productive area to explore, given that previous systematic reviews on the use of digital technology for caregivers of patients with psychosis [92] or dementia [93] have shown positive outcomes.

Our findings show a predominance of text-based conversational agents, with only a few apps using speech as the main mode of communication. Yet, certain populations, such as older people, may be more comfortable interacting via speech, as some individuals may find the dexterity involved with typing on small keypads on smartphones challenging and time consuming. Furthermore, most conversational agents included in our review were app based. Research shows that the use of apps (which need to be downloaded and regularly updated) is often associated with high dropout rates and low utilization [94]. Such disadvantages do not seem to apply to messaging apps such as Facebook Messenger, iMessage, Telegram, WeChat, or WhatsApp, which are already commonly used in the general population. Future research should aim to overcome this limitation brought on by smartphone apps by embedding future health care conversational agents in platforms, which the target population already uses regularly. The advantage of having numerous publishing platform options is the novelty of conversational agents over smartphone apps, and this should be further explored.

A recent systematic review on the effectiveness of ECAs and other conversational agents noted a lack of an established method for evaluating health care conversational agents in health care and a dearth of data on adverse effects [32]. This corresponds to our findings, with most studies being case studies and lacking information on potential adverse effects. Side effects to consider may relate to the content of the conversational agent conversations, which may not be accurate, evidence based, or suitable for the specific circumstance. For example, if a mental health conversational agent user has suicidal tendencies, the conversational agent may not be best equipped to handle such a situation and may provide inappropriate advice, leaving the user at fatal risk. Additional unwanted effects could arise from the black box effect associated with the use of machine learning–based conversational agents, whereby their suggestions are somewhat unpredictable [95]. Furthermore, conversational agents allowing for free text input may lead to significant privacy concerns, especially for vulnerable populations, as individuals can share private and sensitive data in conversations [96]. There is a need for stringent certification from a regulatory board in cases where conversational agents are given roles akin to health care professionals.

The health care sectors for conversational agent application identified in the review were generally very broad, with references to only a few specialties including mental health [97], neurodegeneration [62], metabolic medicine (obesity [47] and diabetes [70,79]), and sexual health [68]. Future applications could expand toward other health care fields where evidence has suggested potential for digital health interventions such as dermatology [98], primary care [99], geriatrics [100], and oncology [101].

There is also a need for more geographically diverse research. Although our review identified 12 articles with a geographical focus in Asia, the evidence stemming from middle-income countries was scarce, and there were no studies from a low-income country. However, digital health initiatives are becoming more common in developing countries, often with a different, context-specific scope, such as ensuring access to health care using social media [102]. To ensure safe and effective use of solutions developed in HIC settings, there is a need for more research to corroborate the safety, effectiveness, and acceptability of these agents in LMICs too. Furthermore, it is important to explore the integration of conversational agents into the existing health systems and services. A *hybrid system*, where digital technology supplements health care services, is increasingly seen as the optimal solution [103]. This mirrors our acknowledgment that conversational agents will be most advantageous in supporting rather than substituting health care professionals. In most studies, conversational agents were developed and presented independently, unsupported by humans, and separate from the existing health care delivery models, which may prove unsustainable in the long run. Future research should consider evaluating *hybrid systems* encompassing conversational agents in their health care delivery, as reported in some of the included studies where conversational agents were complemented by frequent meetings and phone calls with the physicians.

Although the studies reported accuracy, efficacy, effectiveness, and acceptability as outcomes, there were no measurements of cost, efficiency, or how the solution led to improved productivity when used instead of or to augment the work of a health professional. Therefore, it was not possible to ascertain whether the solutions developed were cost-effective compared with alternative approaches.

### Strengths and Limitations

We conducted a comprehensive literature search of multiple databases, including gray literature sources. We prioritized sensitivity over specificity in our search strategy to capture a holistic representation of conversational agent usage uptake in health care. However, given the novelty of the field and the employed terminology, some unpublished studies discussed at niche conferences or meetings may have been omitted. Furthermore, although classification of the themes of our conversational agents was based on thorough analysis, team discussions, and consensus, it might not be all inclusive and may require further development with the advent of new conversational agents. In addition, although some conversational agents belong to more than 1 theme, we mostly classified them based on the dominant mode of application for the sake of clarity. Finally, we excluded articles with poorly reported data on chatbot assessments; therefore, we may have missed some health care conversational agents (Multimedia Appendix 5 [36,97,104-188]). We decided to exclude these because they did not appear to contribute anything additional or noteworthy to our review. The personality traits presented were guided by a reference paper on chatbot personality assignment [43] and also a condensation of descriptive terms from several articles. The lack of depth and breadth in the description of the content and development of many conversational agents led us to organically develop a framework for this paper. This framework is, therefore, still exploratory and adapted to suit the purposes of this review and may well be explored and further refined with more in-depth analysis such as previously published frameworks [189].

### Conclusions

Conversational agents are an up-and-coming form of technology to be used in health care, which has yet to be robustly assessed. Most conversational agents reported in the literature to date are text based, machine learning driven, and mobile app delivered. Future research should focus on assessing the feasibility, acceptability, safety, and effectiveness of diverse conversational agent formats aligned with the target population’s needs and preferences. There is also a need for clearer guidance on health care –related conversational agents’ development and evaluation and further exploration on the role of conversational agents within existing health systems.

## Appendix

Multimedia Appendix 1 Search strategy.

Multimedia Appendix 2 Types of user input (blue) and output (green) in the conversational agents.

Multimedia Appendix 3 Characteristics of conversational agents reported in the included studies.

Multimedia Appendix 4 Characteristics of included studies.

Multimedia Appendix 5 List of excluded studies and reasons for exclusion.

## Abbreviations

AI: artificial intelligence
CBT: cognitive behavioral therapy
ECA: embodied conversational agent
EMBASE: Excerpta Medica database
HIC: high-income country
LMIC: low- and middle-income country
MEDLINE: Medical Literature Analysis and Retrieval System Online
NLP: natural language processing
OCLC: Online Computer Library Center
SAT: structure association technique
