# Supplementary material for: Conversational Agents in Health Care: Scoping Review and Conceptual Analysis
Source: J Med Internet Res. 2020 Aug 7;22(8):e17158. doi: 10.2196/17158 (PMC7442948; doi:10.2196/17158)
Supplement: Multimedia Appendix 1 [file jmir_v22i8e17158_app1.docx]

**Date of Search: 8th April 2019**

**List of keywords**

1. Artificially intelligent chatbot
2. Artificially intelligent chat agent
3. Automated virtual agent
4. Automated chat agent
5. Artificial intelligence chatbot
6. Artificial conversational entity
7. AI agent
8. Chatterbox
9. Chatbot
10. Chatterbot
11. Chat assistant
12. Conversational agent
13. Conversive agent
14. Conversational system
15. Conversational assistant
16. Conversational User Interface
17. Conversational Personal Assistant
18. Conversational interface
19. Conversational avatar
20. Conversational computer
21. Conversational humanoid
22. Conversational Character
23. Conversational bot
24. Conversational AI
25. Cyber individual
26. Dialog system
27. Interactive online character
28. Interactive talking program
29. Interactive virtual agent
30. Interactive agent
31. Interactive conversational assistant
32. Intelligent virtual agent
33. Intelligent virtual assistant
34. Intelligent conversational assistant
35. Intelligent conversational avatar
36. Intelligent agent
37. Online chat agent
38. Smartbot
39. Smart virtual assistant
40. Sociable agent
41. Talk bot
42. Talking agent
43. Talking avatar
44. Text-based healthcare chatbot
45. Text based dialogue system
46. Text-based synchronous chat
47. Virtual chat agent
48. Virtual personal assistant
49. Virtual online assistnat
50. Virtual host
51. Virtual hostess
52. Virtual human
53. Virtual human agent
54. Virtual human avatar
55. Virtual human persona
56. Virtual chat agent
57. Virtual chat expert
58. Virtual coach
59. Virtual consultant
60. Virtual conversational agent
61. Virtual advisor
62. Virtual agent
63. Virtual assistant
