# Supplementary figures and images for: Conversational Agents in Health Care: Scoping Review and Conceptual Analysis
Source: J Med Internet Res. 2020 Aug 7;22(8):e17158. doi: 10.2196/17158 (PMC7442948; doi:10.2196/17158)

**
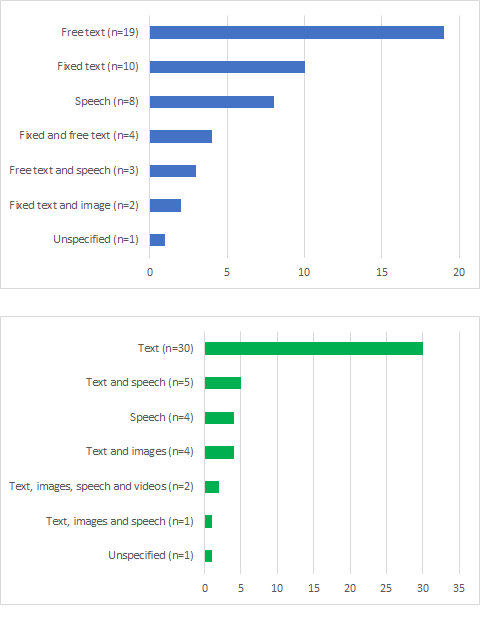
Multimedia appendix 2 – Types of user input (blue) and output (green) in the conversational agents**

Supplement: Multimedia Appendix 2 [file jmir_v22i8e17158_app2.docx]
