# Supplementary material for: Conversational Agents in Health Care: Scoping Review and Conceptual Analysis
Source: J Med Internet Res. 2020 Aug 7;22(8):e17158. doi: 10.2196/17158 (PMC7442948; doi:10.2196/17158)
Supplement: Multimedia Appendix 3 [file jmir_v22i8e17158_app3.docx]

**Multimedia Appendix 3 - Characteristics of conversational agents reported in the included studies**

| **Study ID** | **Conversational agent name** | **Conversational agent delivery channel** | **Conversational Agent Personality** | **Human Involvement** | **Short/Long term goals (Description)** |
| --- | --- | --- | --- | --- | --- |
| Ballati *et al.* (2018) [44] | Siri, Google Assistant, Amazon Alexa | Smartphone embedded software* | NA | NA | Short (To test the CA ability to comprehend individual spoken sentences from the dysarthric user and do so consistently) |
| Bickmore *et al.* (2018) [87] | Siri, Alexa, Google Assistant | Smartphone embedded software* | NA | NA | Short (to provide advice or information for the user’s specific medical question) |
| Casas *et al.* (2018) [52] | Rupert le nutritioniste | Facebook messenger | Coach-like, informal, culture-specific | NA | Long (To encourage long term healthy eating habits - food coaching) |
| Chaix *et al.* (2018) [53] | Vik | Website, app, messenger | Culture-specific, informal | NA | Long (To support patients with breast cancer and their relatives via personalized text messages addressing their queries) |
| Cheng *et al.* (2018) [79] | Healthy Coping with Diabetes | Google Home assistant application (speaker/website) | Human-like | NA | Long (To promote the self-management of type 2 diabetes by older people) |
| Clinical trial NCT03384550 (2017) [51] | Ally | Smartphone based platform (either android or iOS) | NA | NA | Long (to increase physical activity in healthy adults through coaching and incentive provision) |
| Clinical trial NCT (2018) [54] | Vik | Unspecified | NA | NA | Long (to provide long term support for breast cancer patients by responding to any questions they have about their therapeutic management) |
| Comendador *et al*. (2015) [65] | Pharmabot | computer-based platform | Informal, conversational agent identity | No. | Short (to provide information on generic medicine to patients) |
| Crutzen *et al*. (2011) [56] | Bzz | Windows Live Messenger | Informal, knowledgeable | No. | Short & Long. Short = answer adolescent questions regarding sex, drugs and alcohol. Long = aim to increase knowledge in general or influence determinants of behaviour |
| Danda *et al.* (2016) [71] | Vaidya | Smartphone application | Healthcare professional- like | NA | Short (diagnosis is provided based on user's symptoms) |
| Denecke *et al.* (2018) [48] | Ana | Smartphone application | Culture-specific, human-like | NA | Short (Collect a comprehensive patient history via a thorough questionnaire prior to a doctor's appointment) |
| Elmasri *et al.* (2016) [73] | Unspecified | web-based | Healthcare professional-like | NA | Long (Provide an alcohol risk assessment based on user's drinking habits & provides educational content to imbue behavior change to reduce alcohol misuse) |
| Fitzpatrick *et al*. (2017) [80] | Woebot | Facebook Messenger | Coach-like | No. (not during the chat. Encourage users to contain 911 for emergencies) | Long (Cognitive behavioral therapy) |
| Fulmer *et al.* (2018) [81] | Tess | Smartphone application (Fb messenger, Slack, SMS) | Informal | No. | Short (To reduce self-identified symptoms of depression and anxiety in college students) |
| Gaffney *et al*. (2013) [57] | MYLO | Web-based | Healthcare professional-like | NA | Long (To guide patients with problem management) |
| Galescu *et al.* (2009) [82] | CARDIAC | Web-based | NA | No. | Short (To develop a plan-based CA to help chronic care patients look after themselves and provide comprehensive health care monitoring) |
| Ghandeharioun *et al.* (2018) [88] | EMMA | Smartphone application | Human-like | No. | Short (the agent assesses the user’s affect and provides a response/task for the user accordingly) |
| Ghosh *et al*. (2018) [74] | Quro | Web-based | Healthcare professional-like | No. | Short (analyses user's symptoms, infers likely conditions and provides advice on what to do next) |
| Griol *et al.* (2015) [63] | Unspecified | Unspecified | NA | No. | Short (An emotionally sensitive conversational system intended to support patients suffering from chronic pulmonary disease) |
| Griol and Callejas (2016) [62] | Unspecified | Smartphone based (android) platform | Healthcare professional-like | NA | Long (multimodal conversational agent that combines user- preferences as with characteristics about the interaction environment, to improve and personalize the healthcare service provided) |
| Heldt *et al*. (2018) [49] | Mobile coach (tele) | Smartphone application | Coach-like | NA | Long (To encourage an increase in physical activity for overweight individuals) |
| Huang *et al*. (2015) [66] | Teenchat | Web-based | Healthcare professional-like, coach-like | NA | Long (Stress management) |
| Inkster *et al.* (2018) [61] | WYSA | Smartphone application | Informal | NA | Long (to improve user’s self-reported depression symptoms) |
| Joerin *et al.* (2019) [75] | Tess | Smartphone application (Fb messenger, SMS, Amazon Alexa/Google Home) | NA | No. | Short (to provide customized psychological support for caregiving professionals, patients, and family caregiver) |
| Kamita *et al.* (2019) [67] | SAT BOT | Smartphone application (LINE) | NA | No. | Short (stress reduction and motivation maintenance |
| Kobori *et al*. (2018) [68] | Unspecified | Web-based/Facebook messenger | factual, knowledgeable | NA | Short (STI diagnosis) |
| Kocielnik *et al.* (2018) [89] | Reflection companion | SMS/MMS | knowledgeable | NA | Long (promote physical activity) |
| Kowatsch *et al*. (2017) [a] [46] | MobileCoach | Smartphone application | Gender specific, informal, conversational agent identity | Yes. Manual chat channel for direct contact with their health professionals | Long (curb obesity) |
| Kowatsch *et al*. (2017) [b] [30] | MobileCoach | Smartphone application | NA | Yes. Dedicated chat channel for patients and health professionals | Long (curb obesity) |
| L’Allemand *et al.* (2018) [50] | Unspecified | Smartphone application | NA | No. | Long (to motivate overweight participates in a lifestyle intervention including relaxation and activity exercises) |
| Liu *et al.* (2018) [83] | Unspecified | Smartphone application/Web-based | NA | No. | Short (To test the effect of three types of empathic expression—sympathy, cognitive empathy, and affective empathy—on individuals’ perceptions of the service and the conversational agent) |
| Lobo *et al.* (2017) [55] | CARMIE | Smartphone application | NA | No. | Short (to provide the patient with a response to their medication related query) |
| Ly *et al*. (2017) [64] | Shim | Smartphone application | Coach-like | No | Long (to manage mental health issues/stress with positive psychology and CBT) |
| Middleton *et al*. (2016) [58] | Babylon Check | Smartphone application | Healthcare professional-like | No. | Short (automated triaging) |
| Miner *et al*. (2016) [84] | Siri, Google Now, S Voice, Cortana | Smartphone embedded software* | NA | No. (but some referral to human involvement if patient is susceptible to harm) | Short (to provides instant, automated answers to patients’ queries) |
| Mujeeb *et al.* (2017) [69] | Aquabot | Unspecified | NA | No. | Short (diagnosis of achluophobia (fear of darkness) and autism disorder) |
| Nadarzynski *et al*. (2018) [59] | Unspecified | Smartphone application/Web-based | NA | NA | Short (To provide sexual health advice using an automated advice system) |
| Ni *et al.* (2017) [77] | MANDY | Smartphone application | NA | No. | Short (to generate a report for the doctor (prior to consultation) regarding the patient's symptoms and likely causes) |
| Razzaki *et al*. (2018) [60] | Babylon Triage and Diagnostic System | Smartphone application | Knowledgeable | No. | Short (online symptom checker for triage and diagnosis) |
| Rhee *et al.* (2014) [85] | mASMAA | Smartphone application | Informal | Yes. | Long (ongoing symptom monitoring, treatment adherence, and nurturing adolescent-parent partnership) |
| Shaikh *et al.* (2018) [70] | Wellthy diabetes (WD) | Smartphone application | Coach-like | Yes. (personalized coaching from an expert diabetes coach in additional to conversational agent) | Long (behavioral modification for better diabetes management and risk reduction) |
| Stasinaki *et al.* (2018) [47] | Unspecified | Smartphone application | Coach-like | Yes. (One option for direct communication with a health professional) | Long (to promote weight loss and improved ability to perform physical activity for obesity therapy) |
| Stein *et al.* (2017) [86] | Lark Weight Loss health Coach AI (HCA) | Smartphone application | Healthcare professional-like | No. | Long (Continued guidance to promote healthy behavior change through improved meal quality and weight loss for diabetes prevention) |
| Van Heerden *et al.* (2017) [78] | Lwazi (male), Nolwazi (female) | Telegram bot | Human-like, gender specific, | Yes. (upon request or if there is risk of self-harm) | Short (Short counselling session to help the user overcome their fears and concerns to go ahead and get a HIV test) |
| Vita *et al.* (2018) [45] | Doctor Apollo | Telegram bot | NA | n/a | Short & Long (Short = book appointment, medication reminders, long = ongoing support for their HIV management) |
| Wang *et al*. (2018) [72] | Unspecified | WeChat platform | Knowledgeable | Yes. (a human administrator is also involved in conversations, and can instruct and guide participants if needed) | Long (Smoking cessation) |
| Wilson *et al*. (2017) [76] | Siri, Google assistant | Smartphone embedded software | Factual | No. | Short (to answer sexual health related questions) |

* Google API – Application Programme Interface, Google SDK = software development kit, TRIPS = The Rochester Interactive Planning System, XML – Extensible Mark up language
