# Supplementary material for: Conversational Agents in Health Care: Scoping Review and Conceptual Analysis
Source: J Med Internet Res. 2020 Aug 7;22(8):e17158. doi: 10.2196/17158 (PMC7442948; doi:10.2196/17158)
Supplement: Multimedia Appendix 4 [file jmir_v22i8e17158_app4.docx]

**Multimedia appendix 4 – Characteristics of included studies**

| **Reference; study design; location; income** | **Field of study** | **User input/agent output modality** | **Theme; types of participants; number of participants** | **Aim** | **Outcomes and findings (self-reported vs objectively assessed)** |
| --- | --- | --- | --- | --- | --- |
| Ballati et al [44]; case study**;** Italy**;** HIC^a^ | Dysarthria | Free speech/speech | TDV^b^ HS^c^; people with dysarthria (n=17) | To investigate to which extent people with dysarthria can use and be understood by the 3 most common virtual assistants, namely, Siri, Google Assistant, and Amazon Alexa | *Accuracy:* Siri, Google Assistant, and Amazon Alexa performed similarly, with an accuracy of the recognition in the range of 50-60%; objective. |
| Bickmore et al [87]; observational study**;** US^d^**;** HIC | Medical information | Free text, speech/speech, text | EDU^e^; general population (n=54) | To explore the use of conversational assistants to access medical information and assessing the risks of such usage | *Effectiveness:* Of the 168 tasks executed on the conversational agents, 29.2% could have had hazardous outcomes, including 16.1%, which could have resulted in death. Alexa failed most tasks (125/394, 91.9%). Siri had the highest task completion rate (365, 77.6%) but also had the highest likelihood of causing harm for the tested scenarios (27, 20.9%); objective; *Acceptability:* satisfaction was neutral, with a median rating of 4 (IQR^f^ 1-6); self-reported. |
| Casas et al [52]**;** case study**;** Switzerland**;** HIC | Healthy eating | Fixed text/text, image, video | BC^g^; general population (n=36) | To present the design for a conversational agent that aims to coach people on how to improve their food lifestyle | *Effectiveness:* 2 choices are available to the user: (1) reduce his consumption of meat or (2) consume more fruits and vegetables. User tests were conducted with 36 participants, and only 11% of challenges on diary intake were successful with the use of conversational agent. However, there was improved consumption in 65% of cases compared with before the intervention; self-reported. |
| Chaix et al [53]**;** observational study**;** France**;** HIC | Breast cancer | Fixed and free text/text | EDU, HS; patients (n=958) | To evaluate a conversational agent (Vik) designed to improve the quality of life of patients with breast cancer by addressing their fears and concerns. An additional observation was the performance of the conversational agent’s medication adherence function | *Acceptability:* the majority (94%) of the participants were satisfied with the agent’s support; self-reported; *Effectiveness:* Vik increased patient compliance with their treatment (medication adherence) thanks to the reminder function; objective. |
| Cheng et al [79]**;** case study**;** US**;** HIC | Diabetes management | Free speech/speech | EDU, HS, TDV: type 2 diabetic patients (n=10) | To propose a "Healthy coping with diabetes" conversational agent designed to support elderly patients in independently managing their type 2 diabetes | *Acceptability*: the majority (80%) of the participants preferred the intervention, and the users were satisfied with the healthy coping functionalities; self-reported. |
| Clinical trial NCT03384550 [51]; ongoing trial**;** Switzerland; HIC | Physical activity | Free text/text | BC; general population (n=274) | To test the efficacy of a smartphone app with a conversational agent component targeted at increasing physical activity in healthy adults through the use of coaching and incentive provision | N/A^h^ |
| Clinical trial NCT [54]; ongoing trial**;** France**;** HIC | Breast cancer patients’ information | Fixed text/text | HS, EDU, patients with breast cancer (n=150) | To present the ability of the conversational agent Vik to provide responses to breast cancer patients’ questions as a group of multidisciplinary doctors would | N/A |
| Comendador et al [65]; survey questionnaire**;** Philippines; LMIC^i^ | Pediatric generic medicine consultation | Fixed and free text/text | T&M^j^, TDV; parents and children (n=18) | To develop a software application (Pharmabot) that will assist the user in taking the right generic medicine for a certain ailment | *Acceptability*: students rated as “Strongly agree” on user-friendliness and consistency of response and rated as “Agree” on the appropriateness of answer and speed of response regarding the use of Pharmabot. Expert rated as “Agree” on the user-friendliness, appropriateness of answer, speed of response, and consistency of response with Pharmabot; self-reported. |
| Crutzen et al [56]; case study**;** The Netherlands; HIC | Health education | Free text/text | EDU; adolescents (n=737) | To test and evaluate a chat agent designed to “answer adolescents' questions related to sex, drugs and alcohol” | *Acceptability:* the conversational agent was favored (48%) over existing modes of information acquisition including search engines (40%), telephone lines (2%), and others (10%); self-reported. Average duration of chatbot use was 45 min; objective. |
| Danda et al [71]**;** case study**;** India**;** LMIC | Symptom diagnosis | Free speech/text, speech | DG^k^; general population (n=10) | To present a spoken dialogue system Vaidya that can provide a rough diagnosis based on symptoms inputted by the user as free-form speech in real time on both laptop and hand-held devices | *Accuracy:* 86% of the dialogues led to successful completion with correct diagnosis. On average, the system required 3.8 steps to reach the diagnosis; objective. |
| Denecke et al [48]**;** case study**;** Switzerland**;** HIC | Self-anamnesis/taking patient history | Free text/text, speech | HS; patients (n=22) | To outline a self-anamnesis conversational agent that allows users to input their patient history before a doctor’s appointment | *Acceptability:* the participants reported mixed responses regarding ease of use, humanity of conversational agent, and input functionalities; self-reported. |
| Elmasri et al [73]**;** case study**;** Australia**;** HIC | Alcohol misuse in young adults | Fixed and free text/text | EDU; young adults (n=17) | To investigate the suitability of conversational agents for a mental health intervention, specific to alcohol drinking habits assessment in young adults | *Acceptability*: the participants reported higher satisfaction rate with the use of mental health conversational agent. Positive comments included informative knowledge base, simple conversation, quick response time, comprehensible, easy to use, personalized, human like, reliable, accurate, and used layman language; self-reported. |
| Fitzpatrick et al [80]; RCT^l^**;** US**;** HIC | Mental health | Free text/text | T&M; youth with depression and anxiety (n=70) | To test a conversational agent that delivers strategies for self-help using CBT^m^, targeted at university students suffering from anxiety and depression | *Effectiveness:* all (100%) of the participants in the Woebot group endorsed having learnt something new versus three-quarters (77%) of the information in the control group; self-reported; *Acceptability:* participants in the Woebot condition checked in with the bot an average of 12.14 times over the 2-week period; objective. |
| Fulmer et al [81]; RCT**;** US**;** HIC | Mental health | Free text/text | HS, T&M: general population (n=74) | To assess the efficacy of the conversational agent, Tess, to reduce symptoms of depression and anxiety | *Effectiveness:* significant differences were found between the control and intervention groups for symptoms of depression and anxiety at baseline and 2-4 weeks after the intervention; self-reported. |
| Gaffney et al [57]; RCT**;** UK^n^**;** HIC | Mental health | Free text/text | T&M; adults with mental health (n=42) | To test a conversational agent that employs techniques from MOL^o^ therapy to help participants solve their problems | *Effectiveness:* problem solving and helpfulness scores were significantly higher for conversational computer-based agent (MYLO) than ELIZA (control). Distress-lowering scores were significantly lowered for both conditions, with no differential effects between computer programs; self-reported. |
| Galescu et al [82]; case study**;** US**;** HIC | Health monitoring | Free speech/speech | HS, T&M, TDV; patients with heart failure (n=14) | To give an overview of CARDIAC, an intelligent conversational assistant that provides health monitoring for heart failure patients, and to give speech recognition results obtained during a recently completed feasibility evaluation of CARDIAC | *Accuracy:* significant number of errors occurred simply because of inadequate vocabulary coverage in the language models. Overall, the out-of-vocabulary rate was about 3% during the checkup phase. A total of 9 of the 14 participants had word error rates under 21% during the checkup phase. The other 5 subjects had error rates above 30%; objective. |
| Ghandeharioun et al [88]; RCT; US; HIC | Improving well-being | Fixed text/text | EDU, BC; general population (n=39) | To introduce an emotionally intelligent personal assistant for the general population | *Effectiveness:* there was a significant difference in the percentage of positive emotions for EMMA (Emotion Aware mHealth agent) (mean 80.55, SD 3.65) and control (mean 69.08, SD 4.16) conditions. The EMMA group reported a higher percentage of positive emotions and a lower percentage of negative emotions. *Acceptability:* engagement with the agent was also found to be higher in the EMMA condition; self-reported. |
| Ghosh et al [74]; case study**;** Australia**;** HIC | Triage and diagnosis | Free text/text | DG, HS; general population (n=30 clinical scenarios/vignettes) | To test a conversational agent with symptom checker functions and it’s ability to provide a medical summary of the patient based on the input information | *Accuracy:* the conversational agent had a precision rate of 82% in diagnosis and provided a more streamlined alternative to completing patient data entry forms; objective. |
| Griol et al [63]**;** case study**;** Spain**;** HIC | E-therapy (electronic therapy) for patients with chronic respiratory diseases | Free speech/text, speech | HS, T&M, TDV; chronic respiratory patients (n=30) | To develop an emotionally sensitive conversational system for patients suffering from chronic pulmonary disease | *Acceptability:* emotionally sensitive version was rated significantly higher for procuring requested information, interaction rate, and frequency as well as empathy; self-reported. |
| Griol and Callejas [62]; case study**;** Spain**;** HIC | Alzheimer disease | Free text, speech/text, speech, image | T&M; patients with Alzheimer disease (n=25 participants and n=6 caregivers) | To propose a framework that allows developing context-aware multimodal conversational agents that dynamically incorporate user-specific requirements and preferences as well as characteristics about the interaction environment to improve and personalize the service that is provided | *Acceptability:* users preferred a multimodal version of the conversational agent (eg, with tactile sensors) and reported higher satisfaction because of greater flexibility in usage. Caregivers were satisfied with the technical aspects and believed the activities prompted by the agent were relevant and could stimulate cognitive abilities; self-reported. |
| Heldt et al; RCT [49]**;** Switzerland**;** HIC | Overweight management | Fixed text/text | BC; overweight adolescents (n=22) | To explore the use of a text-based health care conversational agent to motivate overweight adolescents to make lifestyle changes such as increased relaxation and physical activity | *Effectiveness:* 43% of adolescents completed their daily challenges successfully. *Acceptability:* 67% of patients had more than 4 interactions with the conversational agent per day within 5.5 months, and an ongoing RCT on the conversational agent indicates high compliance for more than 6 months; objective. |
| Huang et al [66]; case study**;** China**;** LMIC | Mental health | Free text/text | T&M; adolescents (n=10) | To pilot a conversational agent that simulates a virtual companion, which is available to listen to and comfort adolescents in stressful situations | *Accuracy:* conversational agent detected the existence of stress with a precision rate of 78.34%; objective. *Effectiveness:* the study reported that 60% of conversations improved the user’s level of stress; self-reported. |
| Inkster et al [61]; case study; UK; HIC | Mental health | Free text/text | BC, T&M; individuals with self-reported depression (n=129) | To explore the effectiveness of a text-based conversational agent to deliver positive psychology and mental well-being techniques to individuals with self-reported symptoms of depression | *Effectiveness:* improvement in self-reported depression scores (PHQ-9^p^) was significantly higher in the high users group (mean 5.84, SD 6.66) compared with the low users group (mean 3.52, SD 6.15). Effect size was moderate (0.63). An unpaired *t* test was also conducted, and this also produced significant results (*P*=.03). *Acceptability:* 67.7% of users commented that the app experience was helpful and encouraging; self-reported. |
| Joerin et al [75]**;** case study**;** Canada and US**;** HICs | Mental health support | Free text, speech/text, image | HS, T&M, TDV; mental health patients and caregivers (n=675) | To present a conversational agent that provides customized psychological support for caregiving professionals, patients, and family caregivers | *Effectiveness:* emotional support from the conversational agent was found to decrease symptoms of depression and anxiety by 13% and 18%, respectively, while only costing a fraction of the fee for a single therapy session; self-reported (depression & anxiety) and objective (cost calculation). |
| Kamita et al [67]**;** RCT**;** Japan**;** HIC | Mental health (counseling) | Fixed text, image/text, image | HS, T&M, TDV; adults (n=27) | To propose a new system that uses a smartphone and conversational agent to boost user motivation and stress reduction | Effectiveness: the study reported higher scores in the conversational agent course than the web course for self-esteem, anxiety, and depression. *Acceptability:* scores for the technology acceptance model were higher in the chatbot course; self-reported. |
| Kobori et al [68]; diagnostic accuracy study; Japan; HIC | Screening for STI^q^ | Fixed text, image/text | DG; general population (n=70) | To test the feasibility of a novel conversational agent system to diagnose STIs using a smartphone | *Accuracy:* conversational agent had 77.7% accuracy in diagnosing STIs. The accuracy rates of diagnosing gonorrheal urethritis, chlamydial urethritis, syphilis, and condyloma acuminatum were 65%, 70%, 85%, and 95%, respectively; objective. *Effectiveness:* after using conversational agent, 97.7% of patients thought to visit the clinic earlier; self-reported. |
| Kocielnik et al [89]; case study; US; HIC | Physical activity | Free text/text | BC; general population (n=33) | To encourage physical activity in users by employing a deep reflection model with a conversational assistant to induce behavior change | *Acceptability:* response rates were high (96% at baseline, 90% at follow-up; 11 participants answered all questions), insinuating high engagement throughout the study. There was a decline in response length from 170.1 characters in the first week (SD 31.8) to 138.1 characters in the second week (SD 17.0). Furthermore, usage of the system beyond the 2-week study period was an indicator of viability. A total of 16 of the 33 participants opted to continue without any reward; objective. |
| Kowatsch et al [46]; RCT; Switzerland; HIC | Childhood obesity | Fixed text/text | T&M, BC; overweight children (n=15) | To test a text-based health care conversational agent/conversational agent (THCB) for use in a childhood obesity intervention | *Acceptability:* almost 70% of the patients had at least four conversational turns with the THCB per day, and 13 patients reported that they enjoyed using the THCB chat; objective. The findings on attachment bond indicated a high degree of social and emotional relationships at the end of the intervention; self-reported. |
| Kowatsch et al [30]; controlled before and after study**;** Switzerland; HIC | BC | Fixed text/text, image | BC; adolescents (n=11) | To test a rule-based conversational agent employed to coach adolescents on how to manage obesity | *Acceptability:* the acceptability by patients was generally very high. Items measured were perceived ease of use (mean 6.7, SD 0.7; *P*<.001), perceived enjoyment (mean 6.2, SD 1.5; *P*<.01), perceived usefulness (mean 5.9, SD 1.1; *P*<.05), intention to use (mean 5.6, SD 1.4; *P*<.05), and some usability problems; self-reported. |
| L'Allemand et al [50]**;** RCT; Switzerland**;** HIC | Childhood obesity | Fixed text/text | HS, BC; overweight adolescents (n=20) | To test a novel conversational agent-based health app for overweight adolescents and to test whether it can motivate participants to make lifestyle changes that promote relaxation and physical activity | *Acceptability:* 70% of the participants had more than 4 text-based health care conversational turns per day, and 37% of the daily challenges were completed successfully; objective. |
| Liu et al**;** RCT [83]**;** US**;** HIC | Health information advisor | Fixed text/text | HS, T&M, TDV; general population (n=158) | To evaluate a conversational agent that offers both emotional and informational support | *Acceptability:* support with sympathy, affective empathy, and cognitive empathy was more positively evaluated than advice-only support in study 1. Similar findings were reported in study 2. In both studies, expression of sympathy or affective empathy did not increase eeriness compared with advice-only support; self-reported. |
| Lobo et al [55]; questionnaire; Portugal; HIC | Heart medication information | Free speech/text, speech | EDU; heart failure patients (n=11) | To present a conversational medication assistant designed to provide advice and information on medication-related queries for heart failure patients | *Acceptability:* *mean deviation refers to the average of the absolute distance between each data point and the mean.  Average scores were given from 1 to 4 along with mean deviation for usability (3, mean deviation=0.87), coherence (4, mean deviation=0.63), naturalness (3, mean deviation=0.68), quality of information (3, mean deviation=0.74), and HF (heart failure)-directed usability (3, mean deviation=0.80). In general, user engagement with CARMIE (A Conversational Medication Assistant for Heart Failure) was successful. The responses were coherent, short, and easy to understand. The system usability scale score was 88, which was equivalent to a “very good” or “B” rank score; self-reported. |
| Ly et al [64]; RCT; Sweden; HIC | Mental health | Fixed and free text/text | T&M; young adults (n=28) | To study how effective a smartphone-integrated conversational agent could be in conveying advice and education regarding positive psychology and CBT to young adults | *Effectiveness:* there was a significant difference in the score changes of self-esteem (*P*=.024), state-trait anxiety inventory (*P*=.038), and self-rating depression (*P*=.043) in favor of conversational agent course; self-reported. *Acceptability:* the participants showed high engagement during the 2-week long intervention, with an average open app ratio of 17.71 times for the whole period; objective. |
| Middleton et al [58]; diagnostic accuracy study; UK; HIC | Automatic symptom checker | Fixed text/text | DG, HS; general population (n=12 clinicians and n=17 nurses) | To assess the safety, efficacy, and speed of a conversational agent designed for medical triage compared with doctors and nurses using clinical vignettes | *Accuracy:* conversational agent achieved 88.2% accuracy in triaging compared with 75.5% in doctors and 73.5% in nurses. Conversational agent provided a clinically safe outcome in 100% of cases and was faster than health care professionals in 89% of cases; objective. |
| Miner et al [84]; case study; US; HIC | Health education | Free speech/speech | EDU; general population (n=68 phones) | To compare the replies provided by 4 well-known conversational agents (Siri, Cortana, S voice, and Google Now) when asked questions about “mental health, interpersonal violence, and physical health” | *Effectiveness:* Siri responded empathetically for issues concerning depression and physical health, and Cortana responded empathetically for matters involving interpersonal violence; objective. |
| Mujeeb et al [69]**;** case study**;** Pakistan**;** LMIC | Diagnostic conversational agent for achluophobia and autism | Free text/text | DG, HS; patients with autism disorders (n=30) | To evaluate the use of a conversational agent in the diagnosis of achluophobia (fear of darkness) and autism disorder | *Accuracy:* diagnostic accuracy of the conversational agent for diagnosis of achluophobia was 85%, 86.64%, and 87.2% for 3 different age groups (aged 18 to 28 years). Accuracy of autism disorder diagnosis was 88%, 87.6%, and 87.53% for 3 other patient groups (aged 1 to 7 years); objective. |
| Nadarzynski et al [59]; case study; UK; HIC | Sexual health education | Unspecified/unspecified | EDU; general population (n=148) | To employ conversational agents to increase public awareness regarding good practice for sexual health in terms of education and health care services | *Acceptability:* participants’ willingness to use the conversational agent was quite low (40%), and they cited privacy and wanting to interact with physical health care professionals as reasons for their reservations; self-reported. |
| Ni et al [77]; case study; New Zealand; HIC | Automating patient intake | Free text/text | HS, DG; general population (n=11) | To propose a conversational agent that can assist health care staff by automating the patient intake process | *Accuracy:* chatbot was assessed for question and prediction accuracy. Question accuracy=matched questions/expected questions. Prediction accuracy=matched hypotheses from our system/diagnostic hypotheses in guidebook. Question accuracy was 100% for respiratory issues, chest pain, and headache, but only 66.7% for dizziness. Prediction accuracy was 100%, 64%, 25%, and 14% for respiratory issues, chest pain, headache, and dizziness, respectively; objective. |
| Razzaki et al [60]; diagnostic accuracy study; UK; HIC | Diagnosis and health care support | Free text/text | DG, HS; general population (n=7 doctors, 100 vignettes) | To test the safety and efficacy of a web-based symptom checker compared with a human doctor using clinical vignettes | *Accuracy:* the conversational agent was able to produce differential diagnoses with precision (44.4% vs 43.6%) and recall comparable with that of doctors (80% vs 83.9%). *Safety:* the conversational agent provided safer triage recommendation than doctors on average (97.0% vs 93.1%) at the expense of a marginally lower appropriateness (90.0% vs 90.5%); objective. |
| Rhee et al [85]**;** case study**;** US**;** HIC | Asthma management | Free text/text | HS, T&M; adolescents with asthma (n=15 adolescent-parent dyads) | To describe the development and evaluation of an mASMAA^r^ designed to facilitate symptom monitoring, treatment adherence, and adolescent-parent partnership | *Effectiveness:* mASMAA improved awareness of symptoms and triggers, encouraged treatment adherence, promoted a sense of control, and facilitated adolescent-parent partnerships; self-reported. *Acceptability:* average response rates to questions asked by the bot was high (range, 81% to 97%). Generally, response rates in the morning were higher than those at bedtime. Each adolescent on average submitted 19 self-initiated text messages (range, 3 to 38), and symptom-related messages were the most common topic of teen-initiated messages, followed by activity-related and medication-related messages; objective. |
| Shaikh et al [70]**;** before and after study**;** India**;** LMIC | Diabetes management | Free text/text | T&M; diabetic patients (n=102) | To report results that aim to validate Wellthy Diabetes as an agent for diabetes management and risk reduction | *Effectiveness:* 102 participants reported a mean postintervention reduction of −0.49% A_1c_^s^ (95% CI −0.73 to −0.25; *P*=.01) with the use of a conversational agent. A total of 60% (n=61) of the participants achieved a greater reduction in their A_1c_, with a mean reduction of −1.17% A_1c_ (95% CI 0.75 to 1.60); objective. |
| Stasinaki et al [47]**;** pilot study**;** Switzerland**;** HIC | Obesity | Fixed text/text | BC; obese adolescents (n=21) | To test the feasibility of a novel health application game as a therapeutic option for adolescents with obesity | *Effectiveness:* physical performance improved significantly in 5 of 8 tests in the intervention group (agility, flexibility, and strength) and only in 2 tests in the control group (agility and endurance). There was no difference between the groups for BMI; objective. |
| Stein et al [86]**;** longitudinal observation study**;** US**;** HIC | Diabetes prevention | Free text/text, images | BC; overweight and obese adults (n=70) | To evaluate weight loss, changes in meal quality, and app acceptability among users of the Lark, Weight Loss Health Coach AI | *Effectiveness:* the participants reported 2.38% weight loss compared with baseline weight, and percentage of healthy meals increased by 31%; objective. *Acceptability:* the average duration of app use was 15 (SD 1.0) weeks, and users averaged 103 sessions each. The in-app user trust survey had a 100% response rate and positive results, with a satisfaction score of 87 out of 100; self-reported. |
| Van Heerden et al [78]**;** case study**;** South Africa**;** LMIC | HIV counseling | Free text/text | EDU; adults (n=10) | To describe a conversational agent capable of guiding someone through the pretest counseling phase of an HIV test | *Acceptability:* the participants reported high acceptability rate with the use of conversational agent and the majority (6/10) thought that the familiarity with chat interface, avatar image, and appropriate responses made the interaction feel substantially like talking with a human; self-reported. |
| Vita et al [45]**;** before and after study**;** Italy**;** HIC**^‑^** | ART (antiretroviral therapy) adherence and HIV management | Free text/text | HS, T&M; patients (n=34) | To design, develop, and evaluate a digital health care personnel-patient interface for better long-term management of HIV infection | *Acceptability:* the conversational agent was used for a variety of functions: 24.6% to book an appointment, 22% to get results of exams, 20% for therapy, 10.2% for upcoming event updates (appointments), and 13.6% for other activities, for example, start, free interaction, and cancel. The “Medication reminder” function was used on less than 10% of days; objective. |
| Wang et al [72]; RCT; Hong Kong; HIC | Smoking cessation | Free text/text | BC, EDU; adult smokers (n=401) | To assess the performance of a social media–driven conversational agent employed for a smoking cessation program | *Effectiveness:* weekly questionnaire indicated that 38.3% reported not having smoked in the past week, whereas 69.4% reported that the frequency of smoking had been reduced during the past week; self-reported. *Acceptability:* the average number of messages per day for each group (Intervention/Control) was 5.24 when the conversational agent was not activated. This number increased by 61% (average 8.45) when the conversational agent was activated. More than half of the active conversations were initiated by the conversational agent. Content-specific interactions were more effective than scheduled plan in stimulating user activities; objective. |
| Wilson et al [76]; case study; New Zealand; HIC | Sexual health education | Free speech/speech, text, image, video | EDU; general population (n=50 questions) | To assess the quality of information in relation to 50 questions concerning sexual health provided by Siri and Google Assistant compared with a Google search | *Accuracy*: Google search performed better than the 2 digital assistants, providing 72% (36/50) of the best (or equal best) responses for the sexual health questions. Google Assistant provided 50% of best (or equal best responses), better than Siri which only provided 32% of best (or equal best responses); *P*=.04). Google searches also had the lowest outright failure rate, providing no useful response for 8% (4/50) of the questions compared with 12% for Google Assistant and 36% for Siri; objective. |

Outcome measures of focus were categorized as: Effectiveness, Accuracy and Acceptability.

^a^HIC: high-income country.

^b^TDV: technical development.

^c^HS: health care support.

^d^US: Unites States.

^e^EDU: education.

^f^IQR: interquartile rate.

^g^BC: behavior change.

^h^N/A: not applicable.

^i^LMIC: low- and middle-income country.

^j^T&M, treatment and monitoring.

^k^DG: diagnosis.

^l^RCT: randomized controlled trial.

^m^CBT: cognitive behavioral therapy.

^n^UK: United Kingdom.

^o^MOL: methods of levels.

^p^PHQ-9: patient health questionnaire-9.

^q^STI: sexually transmitted infection.

^r^mASMAA: mobile phone–based asthma self-management aid for adolescents

^s^A1c: glycated haemoglobin. Indication of blood sugar levels.
