# Supplementary material for: Conversational Agents in Health Care: Scoping Review and Conceptual Analysis
Source: J Med Internet Res. 2020 Aug 7;22(8):e17158. doi: 10.2196/17158 (PMC7442948; doi:10.2196/17158)
Supplement: Multimedia Appendix 5 [file jmir_v22i8e17158_app5.docx]

**Multimedia Appendix 5 – List of excluded studies and reasons for exclusion**

| **Study ID** | **Title** | **Reason for exclusion** |
| --- | --- | --- |
| Abashev et al. (2017) [101] | Programming Tools for Messenger-Based Chatbot System Organization: Implication for Outpatient and Translational Medicines | No evaluation data* |
| Ahmad et al. (2018) [102] | Conversational bot for pharmacy: A natural language approach | Limited evaluation data** |
| Alexander 1973 [103] | Computer assisted optometry a tutorial with examples | No evaluation data |
| Amato et al. (2017) [104] | Chatbots meet eHealth: automatizing healthcare | No evaluation data |
| Atay et al. (2016) [105] | Can a smartphone-based chatbot engage older community group members? the impact of specialised content | Limited evaluation data** |
| Beun et al. (2016) [106] | Improving adherence in automated e-coaching | No evaluation data |
| Brixey (2017) [107] | SHIHbot: A Facebook chatbot for sexual health information on HIV/AIDS | No evaluation data |
| Callejas et al. (2014) [108] | A virtual coach for active ageing based on sentient computing and m-health | No evaluation data |
| Cameron et al. (2017) [109] | Towards a chatbot for digital counselling | No evaluation data |
| Cameron et al. (2018) [110] | Best Practices for Designing Chatbots in Mental Healthcare–A Case Study on iHelpr | No evaluation data |
| Chung et al. (2018) [111] | Chatbot-based healthcare service with a knowledge base for cloud computing | No evaluation data |
| Cooper at al. (2018) [112] | Designing a Chat-Bot for Non-Verbal Children on the Autism Spectrum | No evaluation data |
| D'Alfonso et al. (2017) [97] | Artificial Intelligence-Assisted Online Social Therapy for Youth Mental Health | No evaluation data |
| Denecke et al. (2018) [113] | Talking to Ana: A Mobile Self-Anamnesis Application with Conversational User Interface | No evaluation data |
| Dharwadkar et al. (2018) [114] | A Medical chatbot | No evaluation data |
| Divya et al. (2018) [115] | A self-diagnosis medical chatbot using artificial intelligence | No evaluation data |
| Do et al. (2016) [116] | Empathic virtual assistant for healthcare information with positive emotional experience | No evaluation data |
| Dubosson et al. (2017) [117] | Going beyond the relapse peak on social network smoking cessation programmes: ChatBot opportunities | No evaluation data |
| Fadhil (2017) (a) [118] | Addressing Challenges in Promoting Healthy Lifestyles | No evaluation data |
| Fadhil (2017) (b) [119] | An adaptive learning with gamification & conversational UIs: The rise of CiboPoliBot | No evaluation data |
| Fadhil et al. (2017) (c) [120] | CoachAI_A conversational UI assisted e-coaching platform | No evaluation data |
| Fadhil (2018) (a) [121] | Beyond Patient Monitoring: Conversational Agents Role in Telemedicine & Healthcare Support For Home-Living Elderly Individuals | No evaluation data |
| Fadhil (2018) (b) [122] | Can a Chatbot Determine My Diet?: Addressing Challenges of Chatbot Application for Meal Recommendation | No evaluation data |
| Fadhil et al. (2018) (c) [123] | A Conversational Interface to Improve Medication Adherence: Towards AI Support in Patient's Treatment | No evaluation data |
| Fadhil et al. (2018) (d) [124] | Assistive Conversational Agent for Health Coaching: A Validation Study | No evaluation data |
| Ferguson (2009) [125] | CARDIAC: An intelligent conversational assistant for chronic heart failure patient heath monitoring | No evaluation data |
| Ferguson (2010) [126] | Towards a Personal Health Management Assistant | No evaluation data |
| Fernandez (2018) [127] | Implementation and feasibility study of a tailored health education bot in Telegram for mothers of children with obesity and overweight | No evaluation data |
| Fischer (2016) [128] | From books to bots: Using medical literature to create a chat bot | No evaluation data |
| Gabrielli (2018) [129] | SLOWBot (chatbot) Lifestyle Assistant | No evaluation data |
| Gatius (2012) [130] | Conversational System to Assist the User when Accessing Web Sources in the Medical Domain | No evaluation data |
| Hassoon (2018) [131] | Increasing Physical Activity Amongst Overweight and Obese Cancer Survivors Using an Alexa-Based Intelligent Agent for Patient Coaching: Protocol for the Physical Activity by Technology Help (PATH) Trial | No evaluation data |
| Hsu (2017) [132] | Allergybot: A chatbot technology intervention for young adults with food allergies dining out | Limited evaluation data** |
| Ireland (2018) [133] | Designing a Chat-Bot for Non-Verbal Children on the Autism Spectrum | No evaluation data |
| Ireland et al. (2015) [134] | Chat-Bots for People with Parkinson's Disease: Science Fiction or Reality? | No evaluation data |
| Ireland et al. (2016) [135] | Hello Harlie: Enabling Speech Monitoring Through Chat-Bot Conversations | Limited evaluation data** |
| Karpagam (2014) [136] | An intelligent conversation agent for health care domain | Limited evaluation data** |
| Kramer (2019) [137] | Investigating Intervention Components and Exploring States of Receptivity for a Smartphone App to Promote Physical Activity: Protocol of a Micro randomized Trial | No evaluation data |
| Kyo-Joong et al. (2018) [138] | Empathy Bot: Conversational Service for Psychiatric Counseling with Chat Assistant | No evaluation data |
| Lee (2017) [139] | The chatbot feels you-a counseling service using emotional response generation | No evaluation data |
| Lokman and Zain (2010) [140] | One-Match and All-Match Categories for Keywords Matching in Chatbot | Limited evaluation data** |
| Lokman et al. (2009) (a) [141] | An architectural design of Virtual Dietitian (ViDi) for diabetic patients | Limited evaluation data** |
| Lokman et al. (2009) (b) [142] | Designing a Chatbot for diabetic patients | No evaluation data |
| Madhu et al. (2017) [143] | A novel approach for medical assistance using trained chatbot | No evaluation data |
| Marciel at al. (2010) [144] | Cell phone intervention to improve adherence: cystic fibrosis care team, patient, and parent perspectives | No evaluation data |
| Marsh (2018) [145] | The use of a chatbot in radiology education | No evaluation data |
| Mascitti et al. (2010) [98] | COACH BOT: Modular e-course with virtual coach tool support | No evaluation data |
| Morris (2018) [146] | Towards an Artificially Empathic Conversational Agent for Mental Health Applications: System Design and User Perceptions | No evaluation data |
| Oh et al. (2017) [147] | A chatbot for psychiatric counseling in mental healthcare service based on emotional dialogue analysis and sentence generation | No evaluation data |
| Oyebode et al. (2018) [148] | Likita: A Medical Chatbot To Improve HealthCare Delivery In Africa | No evaluation data |
| Pereira and Díaz (2018) [149] | Chatbot dimensions that matter: Lessons from the trenches | No evaluation data |
| Rarhi et al. (2017) [150] | Automated medical chatbot | No evaluation data |
| Ricardo et al. (2018) [151] | Proposal for the development of a mobile virtual assistant for treatment of tuberculosis | No evaluation data |
| Rick et al. (2019) [152] | SleepBot: encouraging sleep hygiene using an intelligent chatbot | No evaluation data |
| Rojas-Barahona et al. (2009) [153] | HomeNL: Homecare assistance in natural language. An intelligent conversational agent for hypertensive patients’ management | No evaluation data |
| Rosruen et al. (2019) [154] | Chatbot Utilization for Medical Consultant System | No evaluation data |
| V. Manoj kumar et al. (2016) [155] | Sanative Chatbot For Health Seekers | No evaluation data |
| Vaira et al. (2018) [156] | Mama bot: A system based on ML and NLP for supporting women and families during pregnancy | No evaluation data |
| Valtolina et al. (2018) [157] | Chatbots and conversational interfaces: Three domains of use | Limited evaluation data** |
| Blanson Henkemans et al. (2009) [158] | An online lifestyle diary with a persuasive computer assistant providing feedback on self-management. | No evaluation data. Not a conversational agent.*** |
| Both et al. (2010) [159] | Towards Fully Automated Psychotherapy for Adults: BAS - Behavioral Activation Scheduling via web and mobile phone | No evaluation data. Not a conversational agent. |
| Allen (2006) [160] | Chester: towards a personal medication advisor | Limited evaluation data** |
| Bickmore et al. (2013) [161] | Automated interventions for multiple health behaviors using conversational agents | No evaluation data. Not a conversational agent.*** (ECA) |
| Radziwill et al. (2017) [162] | Evaluating Quality of Chatbots and Intelligent Conversational Agents | No evaluation data |
| Miner et al. (2016) [163] | Conversational agents and mental health: Theory-informed assessment of language and affect | No evaluation data |
| Rizzo et al. (2011) [164] | An intelligent virtual human system for providing healthcare information and support | No evaluation data. Not a conversational agent.*** |
| Shawar et al. (2007) [165] | Different measurements metrics to evaluate a chatbot system | Limited evaluation data** |
| Følstad et al. (2017) [184] | Chatbots and the new world of HCI | Limited evaluation data** |
| Hoermmann et al. (2017) [36] | Application of Synchronous Text Based Dialogue Systems in Mental Health Interventions: Systematic Review | No evaluation data |
| Mishra et al. (2018) [166] | Dr.Vdoc: A Medical Chatbot that Acts as a Virtual Doctor | Limited evaluation data** |
| Kazi et al. (2012) [167] | MedChatBot: An UMLS based Chatbot for Medical Students | Limited evaluation data** |
| Fadhil et al. (2016) [168] | Coach me: a platform for promoting healthy lifestyle | Limited evaluation data** |
| Bickmore et al. (2010) [169] | Usability of conversational agents by patients with inadequate health literacy: evidence from two clinical trials | No evaluation data. Not a conversational agent.*** (ECA) |
| Lindenberg et al. (2011) [170] | E-health for individualized prevention of eating disorders | No evaluation data. Not a conversational agent. |
| Dowling et al. (2016) [171] | Exploring hope and expectations in the youth mental health online counselling environment | No evaluation data. Not a conversational agent. |
| Dowling et al. (2014) [172] | Investigating individual online synchronous chat counselling processes and treatment outcomes for young people | No evaluation data. Not a conversational agent. |
| Dowling et al. (2015) [173] | A naturalistic study of the effects of synchronous online chat counselling on young people's psychological distress, life satisfaction and hope | No evaluation data. Not a conversational agent. |
| Azevedo et al. (2018) [174] | Using conversational agents to explain medication instructions to older adults | No evaluation data. Not a conversational agent.*** (ECA) |
| Brown et al. (1981) [175] | A conversational information computer system for health and safety operation: The occupational surveillance interactive system (OSIS) | No evaluation data. Not a conversational agent.*** |
| Crutzen et al. (2014) [176] | What can we learn from a failed trial: insight into non-participation in a chat-based intervention trial for adolescents with psychosocial problems | No evaluation data. Not a conversational agent.*** |
| Denecke et al. (2019) [177] | Intelligent Conversational Agents in Healthcare: Hype or Hope? | No evaluation data |
| Fadhil et al. (2019) [178] | Designing for Health Chatbots | Limited evaluation data** |
| Yang et al. (2016) [179] | Mindbot: A Social-Based Medical Virtual Assistant | No evaluation data |
| Stieger et al. (2018) [180] | PEACH, a smartphone- and conversational agent-based coaching intervention for intentional personality change: study protocol of a randomized, wait-list controlled trial | No evaluation data |
| Palanica et al. (2019) [181] | Physicians’ Perceptions of Chatbots in Health Care: Cross-Sectional Web-Based Survey | No evaluation data |
| Moura et al. (2018) [182] | Intelligent chatbot for analysis and diagnosis of the psychiatric disorders | No evaluation data |
| Kowatsch et al. (2018) [183] | Designing Just-in-time Adaptive Interventions and Healthcare Chatbots with the Open Source Platform MobileCoach | Limited evaluation data** |

***No evaluation data:**

- Papers with no health-related outcomes tested or presented. We only report on health-related outcomes. Details on technical outcomes are beyond the scope of this paper
- Some literature in the format of posters or protocols for example, do not present any evaluation data

****Limited evaluation data:**

eg. Limited qualitative data presented, with no supporting quantitative findings.

*****No evaluation data. Not a conversational agent:**

In some papers, the agent described (eg. ECA) did not match our definition of a conversational agent in this paper (ie. A non-embodied agent using text, speech or images as the main modality of communication with users).
